# *Boc* modifies the spectrum of holoprosencephaly in the absence of *Gas1* function

**DOI:** 10.1242/bio.20147989

**Published:** 2014-07-25

**Authors:** Maisa Seppala, Guilherme M. Xavier, Chen-Ming Fan, Martyn T. Cobourne

**Affiliations:** 1 Department of Craniofacial Development and Stem Cell Biology, King's College London Dental Institute, Guy's Hospital, London SE1 9RT, UK; 2 Department of Orthodontics, King's College London Dental Institute, Guy's Hospital, London SE1 9RT, UK; 3 Department of Embryology, Carnegie Institution of Washington, Baltimore, MD 21218, USA

**Keywords:** Boc, Sonic hedgehog, Gas1, Lobar holoprosencephaly, Cleft lip and palate, Apoptosis

## Abstract

Holoprosencephaly is a heterogeneous developmental malformation of the central nervous system characterized by impaired forebrain cleavage, midline facial anomalies and wide phenotypic variation. Indeed, microforms represent the mildest manifestation, associated with facial anomalies but an intact central nervous system. In many cases, perturbations in sonic hedgehog signaling are responsible for holoprosencephaly. Here, we have elucidated the contribution of Gas1 and an additional hedgehog co-receptor, Boc during early development of the craniofacial midline, by generating single and compound mutant mice. Significantly, we find *Boc* has an essential role in the etiology of a unique form of lobar holoprosencephaly that only occurs in conjunction with combined loss of *Gas1*. Whilst *Gas1^−/−^* mice have microform holoprosencephaly characterized by a single median maxillary central incisor, cleft palate and pituitary anomalies, *Boc^−/−^* mice have a normal facial midline. However, *Gas1^−/−^*; *Boc^−/−^* mutants have lobar holoprosencephaly associated with clefting of the lip, palate and tongue, secondary to reduced sonic hedgehog transduction in the central nervous system and face. Moreover, maxillary incisor development is severely disrupted in these mice, arresting prior to cellular differentiation as a result of apoptosis in the odontogenic epithelium. Thus, Boc and Gas1 retain an essential function in these tooth germs, independent of their role in midline development of the central nervous system and face. Collectively, this phenotype demonstrates both redundancy and individual requirements for *Gas1* and *Boc* during sonic hedgehog transduction in the craniofacial midline and suggests *BOC* as a potential digenic locus for lobar holoprosencephaly in human populations.

## INTRODUCTION

Holoprosencephaly (HPE) is a surprisingly common developmental field defect affecting the central nervous system (CNS), which is characterized by a failure of the embryonic forebrain to divide in an appropriate manner (Geng and Oliver, 2009; Muenke and Beachy, 2000). Classically, HPE has been divided into alobar, semilobar and lobar forms, which collectively describe the amount of cleavage within the telencephalon (Cohen, 2006); whilst more recently, middle inter-hemispheric fusion and septopreoptic variants have also been reported (Hahn and Barnes, 2010). In the most severe alobar form, the brain has only a single cerebral hemisphere, lacks any inter-hemispheric division, and has an absence of the corpus callosum and olfactory bulbs. In the majority of cases, HPE is accompanied by facial dysmorphogenesis, which also ranges in severity. In the worst manifestation, there is frank cyclopia and the presence of a large superiorly positioned midline proboscis, which dominates a cyclopic and rudimentary face (Cohen, 2006). However, other facial anomalies associated with HPE can be less severe and include the presence of ocular hypotelorism, single nostril, premaxillary agenesis, cleft lip and palate, philtral dysgenesis and single median maxillary central incisor (SMMCI) (DiBiase and Cobourne, 2008). Microform HPE is a specific variant of this condition, associated with facial anomalies that are characteristically at the milder end of the spectrum and occur in the presence of normal development and function within the CNS (Solomon et al., 2012).

The HPE spectrum has a complex etiology, with both genetic and environmental factors having been implicated (Helms et al., 2008). In humans, most cases of HPE are sporadic, caused by chromosomal abnormalities or syndromic disorders and generally incompatible with life (Kauvar and Muenke, 2010). Those individuals that do survive generally have non-syndromic HPE, with fourteen dominant loci currently identified in association with these forms, collectively encoding proteins functioning within four of the major molecular signaling pathways (Bone Morphogenetic Protein, Fibroblast Growth Factor, Nodal and Hedgehog) (Bae et al., 2011; Roessler and Muenke, 2010). However, environmental factors are also implicated in HPE and can include maternal diabetes, alcohol ingestion, cholesterol-lowering drugs and the steroidal alkaloid cyclopamine (Cohen, 1989; Cohen and Shiota, 2002). A key feature of non-syndromic autosomal dominant HPE is the marked clinical variation that is seen, with notoriously poor genotypic–phenotypic correlation and wide-ranging intra-familial variability (Ming and Muenke, 2002). This almost certainly reflects the complex temporo-spatial interplay that occurs between multiple signaling pathways during early morphogenesis of the forebrain and facial region. Indeed, there is evidence from some pedigrees that phenotype is influenced not only by the type of mutation but also the number, with multigenic inheritance being identified in some cases (Ming and Muenke, 2002). Coupled with a growing list of environmental factors associated with HPE, this is indicative of a complex multifactorial disorder.

Amongst the genetic components implicated in the etiology of HPE, disruptions within the Sonic hedgehog (Shh) signaling pathway are known to play a key role. Shh is an important early midline signal within the developing CNS, involved in reiterative patterning of the early neural plate and neural tube, including the forebrain through signaling from the prechordal mesendoderm, rostral diencephalon and telencephalon (Hébert and Fishell, 2008; Wilson and Rubenstein, 2000). Moreover, *Shh* is also expressed in epithelium of the early frontonasal and maxillary processes and makes a significant contribution to patterning of the frontonasal region within the face (Marcucio et al., 2011). Shh signaling is mediated through binding of ligand to the Patched-1 (Ptch1) transmembrane receptor, which leads to de-repression of a G protein-coupled receptor Smoothened (Smo) and pathway activation through modification of Gli transcription factor activity (Briscoe and Thérond, 2013; McMahon et al., 2003). Mice with targeted disruption of *Shh* have alobar HPE and cyclopia (Chiang et al., 1996); whilst in humans, mutation in a number of pathway components have been associated with various forms of HPE, including *SHH* itself (Belloni et al., 1996; Roessler et al., 1996), *PTCH1* (Ming et al., 2002), the upstream transmembrane protein DISPATCHED-1 (*DISP1*) (Roessler et al., 2009) and the downstream transcription factor *GLI2* (Roessler et al., 2003). More recently, a number of co-receptors for Shh have been identified in the mouse, which include the GPI-linked membrane glycoprotein Growth arrest-specific 1 (Gas1) (Martinelli and Fan, 2007) and the closely related Ig/fibronectin single-pass membrane-spanning cell adhesion proteins Cdon (cell adhesion associated, oncogene regulated) and Boc (Boc cell adhesion associated, oncogene regulated) (Kang et al., 1997; Kang et al., 2002). Gas1, Cdon and Boc are able to interact directly with Shh (Lee et al., 2001a; Martinelli and Fan, 2007; McLellan et al., 2008; Okada et al., 2006; Tenzen et al., 2006) and form high-affinity individual complexes with Ptch1 on the surface of receiving cells (Bae et al., 2011; Izzi et al., 2011). Collectively, these three co-receptors demonstrate a co-operative and obligatory role during Shh signaling. Significantly, loss-of-function associated with *GAS1* and *CDON* have both been associated with HPE in humans (Bae et al., 2011; Ribeiro et al., 2010) and mice (Allen et al., 2007; Cole and Krauss, 2003; Martinelli and Fan, 2007; Seppala et al., 2007; Tenzen et al., 2006; Zhang et al., 2011; Zhang et al., 2006) (supplementary material Table S1); whilst *Boc* mutant mice lack HPE but do have misguided commissural axon guidance, cerebellum reduction and reduced ipsilateral retinal ganglion cells (Izzi et al., 2011; Okada et al., 2006; Sánchez-Arrones et al., 2013).

We are interested in the molecular mechanisms that contribute to the phenotypic heterogeneity that characterizes midline facial anomalies seen in HPE and in particular, the role of Shh signaling. Here, we have further elucidated the relative contributions of Gas1 and Boc co-receptor function in HPE using single and compound mutant mice. Significantly, we find evidence of an essential role for *Boc* in the etiology of a unique form of lobar HPE that occurs in the combined absence of *Gas1*. These findings suggest that *BOC* represents an additional potential locus for HPE in human populations.

## MATERIALS AND METHODS

### Generation and genotyping of *Gas1*, *Cdon* and *Boc* mutant and compound mutant mice

All mice were housed and all experiments conducted in compliance with the approved protocols at King's College London, UK and the Carnegie Institution of Washington, USA. *Gas1^−/−^* mutant mice were generated and maintained in a 129sv/C57BL6 mixed background and genotyped as previously described (Martinelli and Fan, 2007). *Cdon^−/−^* and *Boc^−/−^* mice were generated and maintained in a CD1/129sv mixed background and genotyped as previously described (Okada et al., 2006). *Gas1^+/−^* mice were crossed with *Cdon^+/−^* or *Boc^+/−^* mice, to generate *Gas1*; *Cdon* and *Gas1*; *Boc* compound mutants, respectively, in a mixed (129sv/C57BL/6/CD1) background. Timed-matings were set up such that noon of the day on which vaginal plugs were detected was considered as embryonic day (E) 0.5.

In this mixed background *Gas1^−/−^*; *Boc^+/−^* mice were infertile and the yield of *Gas1^−/−^*; *Boc^−/−^* embryos was significantly below that predicted by Mendelian ratios. At E14.5 we obtained 4 *Gas1^−/−^*; *Boc^−/−^* mice from a total of 136 embryos. Interestingly, the yield of *Gas1^+/−^*; *Boc^−/−^* and *Gas1^−/−^*; *Boc^+/−^* was also reduced at E14.5 (n = 8/136 and n = 9/136 embryos, respectively).

### Histological and skeletal analysis

For histological analysis, embryos were fixed in 4% paraformaldehyde (PFA) at 4°C, dehydrated through a graded ethanol series, embedded in paraffin wax, sectioned at 7 µm and stained with haematoxylin and eosin. For differential staining of bone and cartilage, E18.5 mice were fixed overnight in 95% ethanol, skinned and eviscerated. Cartilage staining was carried out in a solution of 76% ethanol, 20% glacial acetic acid and 0.015% alcian blue 8GX (Sigma–Aldrich) for 24 hours, differentiating for 7 days in 95% ethanol, macerating in 1% KOH for 24 hours and washing overnight under running tap water. Bone staining was carried out by transferring the heads to a freshly made 0.1% aqueous solution of alizarin red S (Sigma–Aldrich), with the addition of several drops 1% KOH to enhance darkness of the red colour. The samples were then washed for 30 minutes under running tap water, decolorized in 20% glycerol in 1% KOH for 1–2 weeks and prepared for storage in increasing concentrations of glycerol in 70% ethanol to a final concentration of 100% glycerol. Skeletal preparations were photographed in light field, submerged in 100% glycerol using a Leica stereomicroscope.

### In situ hybridisation

Radioactive section in situ hybridisation was carried out as previously described (Wilkinson, 1992). Light and dark-field images of sections were photographed using a Zeiss Axioscop microscope and merged in Adobe Photoshop CS.

### Proliferation assay

Bromodeoxyuridine (BrdU) labeling for cell proliferation was carried out on histological sections using a Zymed BrdU Labeling and Detection Kit (Invitrogen) according to the manufacturer's instructions. Mouse embryos were labeled with BrdU via intra-peritoneal injection into pregnant females (5 mg/100 g body weight) 2 hours prior to sacrifice.

### Apoptosis

Immunohistochemical detection of apoptotic cell death was carried out on histological sections (prepared as described above) using Terminal deoxynucleotidyl transferase-mediated deoxyUridine triPhosphate Nick End Labeling (TUNEL). TUNEL was carried out using an APOPTag® Plus Fluorescein In Situ Apoptosis Detection Kit (Chemicon International) according to the manufacturer's instructions.

## RESULTS

### Shh co-receptors are differentially expressed in the early frontonasal region

**Fig. 1.**
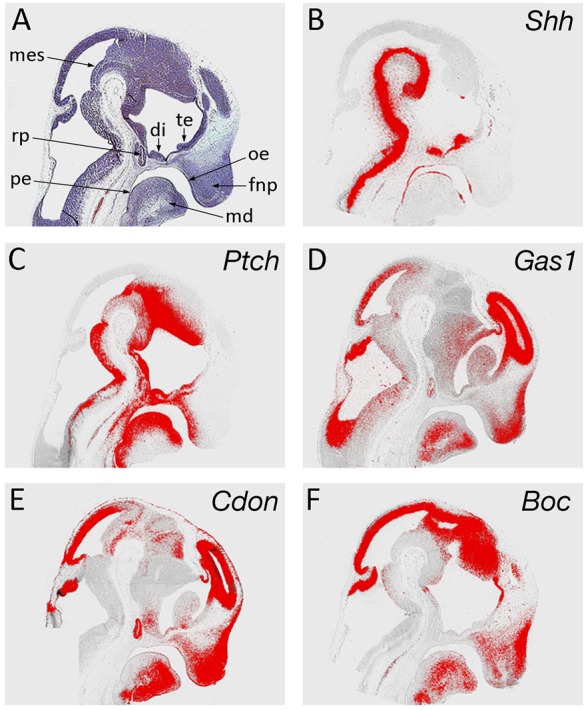
Shh receptor gene expression in the FNP at E11.5. (A) Haematoxylin and eosin; (B) *Shh*; (C) *Ptch1*; (D) *Gas1*; (E) *Cdon*; (F) *Boc*. di, diencephalon; fnp, frontonasal process; md, mandibular process; mes, mesencephalon; oe, oral epithelium; pe, pharyngeal endoderm; rp, Rathke's pouch; te, telencephalon.

To further define the requirements for Shh reception during early facial development, we analyzed the expression domains of *Ptch1*, *Gas1*, *Cdon* and *Boc* in the frontonasal process (FNP) of the mouse embryo and compared them with Shh signaling activity (Fig. 1A–F). At E11.5, Shh is produced in epithelium of the facial midline with signaling marked by a gradient of *Ptch1* expression extending from this source into the underlying mesenchyme of the FNP (Fig. 1B,C). This reflects a requirement for appropriate Shh signal levels during normal development of this region (Marcucio et al., 2011). At this stage, *Gas1* expression is largely reciprocal to *Ptch1* (Seppala et al., 2007), consistent with *Gas1* as a negative transcriptional target of high-level Shh signaling (Fig. 1D) (Allen et al., 2007; Martinelli and Fan, 2007). *Cdon* and *Boc* expression broadly mirrors that of *Gas1* in the FNP, extending in a gradient originating from the periphery of the Shh signaling domain, and also consistent with negative regulation of both these genes by Shh (Tenzen et al., 2006). However, subtle differences do exist, with *Cdon* and *Boc* both demonstrating broader domains than *Gas1*, and *Cdon* having a greater intensity of expression overall (Fig. 1E,F). Interestingly, *Gas1*, *Cdon* and *Boc* all show high-level and overlapping expression in the outer region of the FNP.

### Gradation of phenotype in the facial midline of Gas1, Cdon and Boc mutant mice

**Fig. 2.**
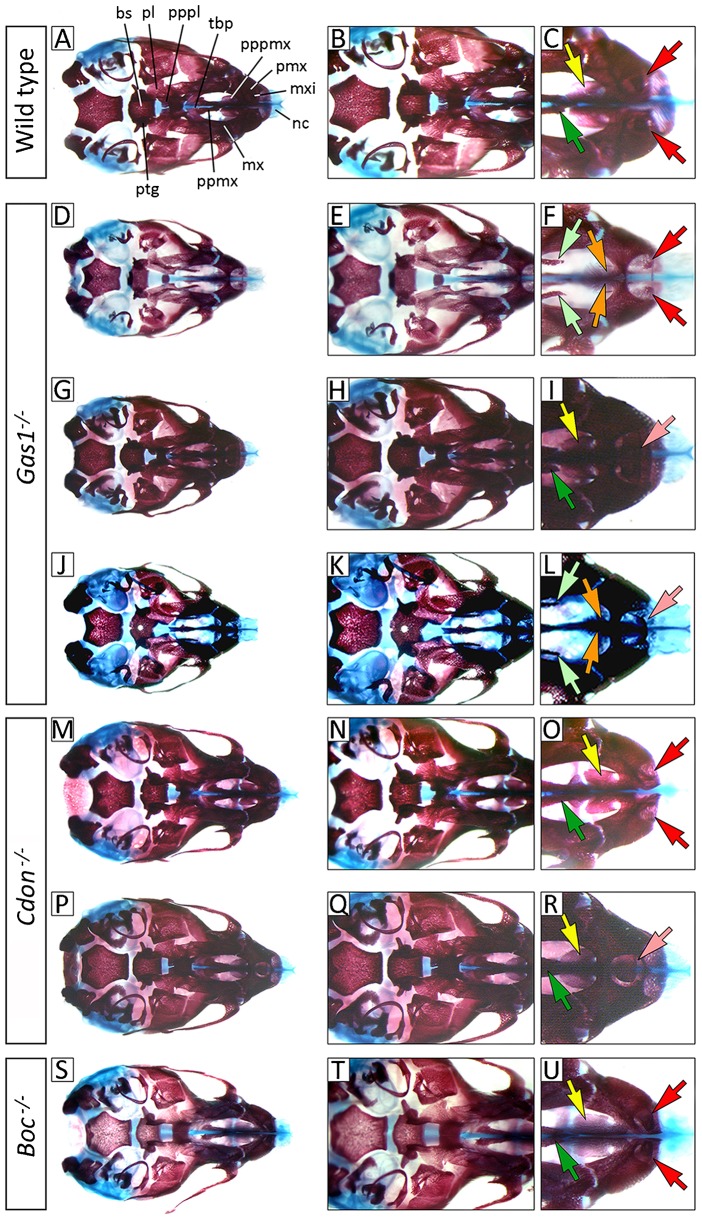
Midline craniofacial defects in the absence of *Gas1* and Cdon but not *Boc*. Comparison of E17.5 wild-type, *Gas1^−/−^* and *Boc^−/−^* cranial base anatomy (norma basalis, mandible removed) differentially stained for bone (alizarin red) and cartilage (alcian blue). (A–C) In wild-type, the premaxillary region consists of the paired premaxillary bones containing the maxillary incisor teeth and the associated palatal processes of the premaxilla. (D–L) *Gas1^−/−^* mice are associated with a spectrum of anomalies affecting the craniofacial midline, including progressive restriction of the incisor field and SMMCI, cleft palate and fenestration of the basisphenoid. (M–R) *Cdon1^−/−^* mice are also associated with midline craniofacial defects including SMMCI; however, the primary and secondary palate remains intact. (S–U) *Boc^−/−^* mice have normal development of the craniofacial midline. Red, yellow and dark green arrows indicate normal development of the paired maxillary incisors, premaxillary and maxillary palatal shelves, respectively; orange and light green arrows indicate hypoplasia of the premaxillary and maxillary palatal shelves, respectively; pink arrows indicate SMMCI. bs, basisphenoid; mx, maxilla; mxi, maxillary incisors; nc, nasal capsule; pl, palatine; pmx, premaxilla; ppmx, palatal process of maxilla; pppl, palatal process of palatine; pppmx, palatal process of premaxilla; ptg, pterygoid plate; tbp, trabecular basal plate.

The domains of *Gas1*, *Cdon* and *Boc* expression in the early FNP suggest a collective requirement for these co-receptors during formation of the early face. To further characterize the role of these co-receptors during development of the craniofacial midline we investigated mutant mice using skeletal preparation at E17.5 (Fig. 2A–U). In a 129sv/C57BL/6/CD1 mixed background, *Gas1^−/−^* mice had a characteristically variable microform HPE, which ranged from essentially normal development, through submucous cleft palate and approximation of the incisor field, to frank midline cleft palate and SMMCI in the worst affected (Fig. 2D–L), which is consistent with those previously reported in a 129sv/C57BL/6 background (Seppala et al., 2007). *Cdon^−/−^* mice are also known to have HPE, including both semilobar and microforms, depending upon background (Cole and Krauss, 2003; Hong and Krauss, 2012; Zhang et al., 2006). In the same mixed background, we found microform HPE associated with an intact lip philtrum and palate, with variable separation of the incisor field (Fig. 2M–R). This differs from a 129sv/C57BL/6 background, which produces microform HPE associated with premaxillary agenesis and philtral dysgenesis (Cole and Krauss, 2003). However, in contrast to *Gas1* and *Cdon*, *Boc^−/−^* mice do not display HPE on any background, including the mixed background investigated here and have normal gross development of the craniofacial midline (Fig. 2S–U) (Okada et al., 2006; Zhang et al., 2011).

### *Boc* interacts with *Gas1* during midline craniofacial development

Given this apparent variation in individual requirements for *Gas1*, *Cdon* and *Boc* during craniofacial development, we further investigated the role of these co-receptors using skeletal preparation of compound mutants.

**Fig. 3.**
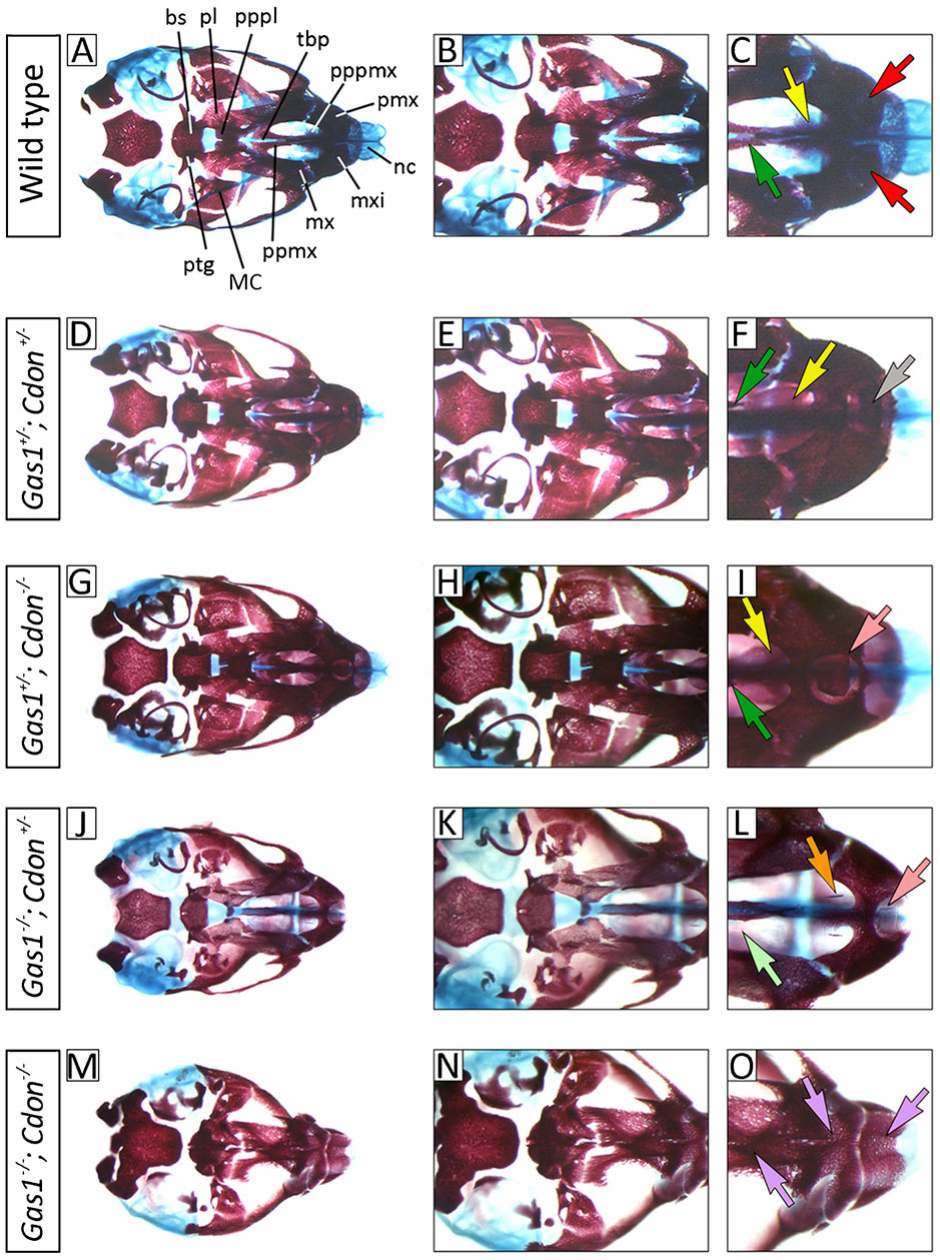
Midline craniofacial defects in *Gas1*; *Cdon* compound mutant mice. Comparison of E17.5 wild-type and *Gas1*; *Cdon^−/−^* compound mutant cranial base anatomy (norma basalis, mandible removed) differentially stained for bone (alizarin red) and cartilage (alcian blue). (A–C) Wild-type mice. (D–F) *Gas1^+/−^*; *Cdon^+/−^* mice have microform HPE characterized by varying degrees of maxillary incisor fusion. (G–I) *Gas1^+/−^*; *Cdon^−/−^* mice have SMMCI. (J–L) *Gas1^−/−^*; *Cdon^+/−^* mice have SMMCI and cleft palate. (M–O) *Gas1^−/−^*; *Cdon^−/−^* mice have significant disruption along the cranial base, including ossification within the nasal capsule and stenosis of the premaxilla. Red, yellow and dark green arrows indicate normal development of the paired maxillary incisors, premaxillary and maxillary palatal shelves, respectively; orange and light green arrows indicate hypoplasia of the premaxillary and maxillary palatal shelves, respectively; grey arrow indicates maxillary incisor fusion; pink arrows indicate SMMCI; violet arrows indicate stenosis within the premaxilla. bs, basisphenoid; MC Meckel's cartilage; mx, maxilla; mxi, maxillary incisors; nc, nasal capsule; pl, palatine; pmx, premaxilla; ppmx, palatal process of maxilla; pppl, palatal process of palatine; pppmx, palatal process of premaxilla; ptg, pterygoid plate; tbp, trabecular basal plate.

*Gas1*; *Cdon* mutant mice on a 129sv; C57BL/6 background have a severe form of HPE, characterized by a lack of medial facial structures affecting both maxilla and mandible (Tenzen et al., 2006). The effect of losing different combinations of *Gas1* and *Cdon* alleles in a mixed 129sv; C57BL/6; CD1 background was similar, although severity of the maxillary and mandibular skeletal aplasia was reduced (Fig. 3D–O). Whilst heterozygous single mutants were normal, *Gas1^+/−^*; *Cdon^+/−^* mice had varying degrees of incisor fusion and premaxillary truncation but the palate remained intact (Fig. 3D–F). Interestingly, in *Gas1^+/−^*; *Cdon^−/−^* mice, frank SMMCI was present but normal development of the palate still occurred (Fig. 3G–I); however, in *Gas1^−/−^*; *Cdon^+/−^* mutants SMMCI was seen in combination with cleft palate (Fig. 3J–L). These data are consistent with findings in single mutants, where there is reliance upon *Gas1* function during palatogenesis but not *Cdon*. Complete loss of *Gas1* and *Cdon* was associated with a much more severe phenotype (Allen et al., 2007), which included HPE and the presence of a single external nares, ossification within the nasal capsule, synostosis and marked truncation of the midface and mandible, and disruption to the secondary palate (Fig. 3M–O).

**Fig. 4.**
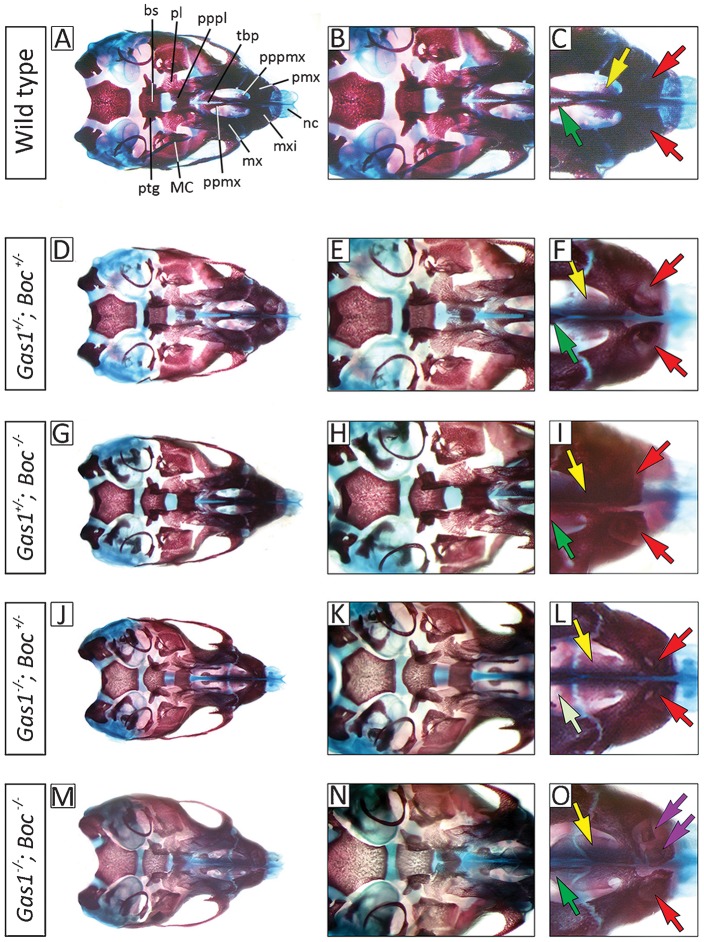
Midline craniofacial defects in *Gas1*; *Boc* compound mutant mice. Comparison of E17.5 wild-type and *Gas1*; *Boc* compound mutant cranial base anatomy (norma basalis, mandible removed) differentially stained for bone (alizarin red) and cartilage (alcian blue). (A–C) Wild-type; (D–F) *Gas1^+/−^*; *Boc^+/−^* and (G–I) *Gas1^+/−^*; *Boc^−/−^* mice all have normal craniofacial anatomy; (J–L) *Gas1^−/−^*; *Boc^+/−^* mice retain the features of *Gas1^−/−^* mice, here there is cleft palate but the maxillary incisors are paired; (M–O) *Gas1^−/−^*; *Boc^−/−^* mice have disrupted maxillary incisor development, with apparent duplication within the premaxilla. Red, yellow and dark green arrows indicate normal development of the maxillary incisor, premaxillary and maxillary palatal shelves, respectively; deep purple arrows indicate duplicated maxillary incisor tooth germs; light green arrows indicate hypoplasia of the maxillary palatal shelves. bs, basisphenoid; MC, Meckel's cartilage; mx, maxilla; mxi, maxillary incisors; nc, nasal capsule; pl, palatine; pmx, premaxilla; ppmx, palatal process of maxilla; pppl, palatal process of palatine; pppmx, palatal process of premaxilla; ptg, pterygoid plate; tbp, trabecular basal plate.

In contrast to *Gas1*; *Cdon* compound mutants, we found grossly normal craniofacial anatomy in *Gas1^+/−^*; *Boc^+/−^* and *Gas1^+/−^*; *Boc*^−/−^ mice and, consistent with the phenotype of *Gas1*^−/−^ mutants, microform HPE in *Gas1^−/−^*; *Boc^+/−^* mice (Fig. 4D–F, Fig. 4G–I, Fig. 4J–L, respectively). Interestingly, in *Gas1^−/−^*; *Boc^−/−^* mutants we found some variation in the premaxillary region that had not previously been described in either *Gas1* (or *Cdon*) single or compound mutants – in particular, the seeming presence of supernumerary or duplicated incisor formation within the premaxilla (Fig. 4M–O).

**Fig. 5.**
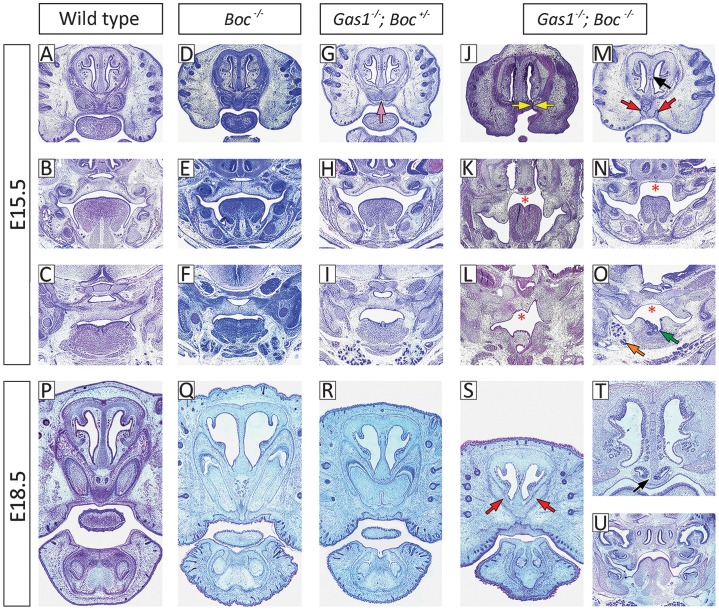
Loss of *Gas1* and *Boc* exacerbates the midline craniofacial defects seen in the absence of *Gas1*. Comparison of the craniofacial midline at E15.5 (A–O) and E18.5 (P–U) in (A–C,P) wild-type; (D–F,Q) *Boc*^−/−^; (G–I,R) *Gas1^−/−^*; *Boc^+/−^* and (J–O,S–U) *Gas1^−/−^*; *Boc^−/−^*. In *Boc^−/−^* mice development of the craniofacial midline is grossly normal, whilst *Gas1^−/−^*; *Boc^+/−^* mice have midline defects characteristic of *Gas1^−/−^* mice (pink arrow indicates maxillary incisor fusion; although note that in this example (H,I) the palate is intact). In *Gas1^−/−^*; *Boc^−/−^* mice, early incisor development is disorganized (red arrows in panel M), with a lack of cellular differentiation and hard tissue formation at later stages (red arrows in panel S). In addition, there is cleft lip (yellow arrows in panel J) and cleft palate associated with a failure of palatal shelf elevation (red asterisk in panels K,N,L,O), clefting of the pharyngeal tongue (green arrow in panel O), superficial positioning of the submandibular gland (orange arrow in panel O), fenestrations in the nasal septum (black arrow in panel M) and abnormal positioning of the vomeronasal organs (black arrow in panel T). All sections stained with haematoxylin and eosin. At E15.5, frontal sections are shown at the level of the maxillary incisors (upper row), first molars (middle row) and pharyngeal region (lower row). At E18.5, sections are through the maxillary incisors (P–S), nasal septum (T) and molar region (U).

We further investigated this phenotype using histological analysis (Fig. 5A–U). At E15.5, the craniofacial midline of *Boc^−/−^* mice was indistinguishable from wild-type, with normal development of the incisors, nasal cavity and palate (Fig. 5A–F); whilst *Gas1^−/−^*; *Boc^+/−^* mutants had a similar phenotype to *Gas1^−/−^* mice, having reduced craniofacial dimensions, cleft palate and SMMCI with variable penetrance. However, these teeth had normal cellular organization and differentiation, with the formation of appropriate hard tissues at the crown stage of development, even in the presence of fusion (Fig. 5G–I). Importantly, *Gas1^−/−^*; *Boc^−/−^* mice revealed a number of significant defects within the craniofacial midline, never previously observed in *Gas1^−/−^* single mutants. In particular, these mice had unilateral cleft lip and a marked increase in severity of the cleft palate, associated with hypoplasia and failed elevation of the palatal shelves. In the pharyngeal region, the tongue dorsum was hypoplastic and cleft in the midline, with hyperplasia of the median circumvallate papillae and superficially-positioned, asymmetric submandibular salivary glands. Moreover, in the nasal cavity there were fenestrations within the nasal septum (Fig. 5J–O). Further examination confirmed the presence of disrupted maxillary incisor development, with these teeth having abnormal tissue architecture associated with irregular budding of the dental lamina and apparent developmental arrest (Fig. 5J,M). At E18.5, in comparison to wild-type, *Boc^−/−^* and *Gas1^−/−^*; *Boc*^+/−^ mice there was no evidence of any histodifferentiation within the *Gas1^−/−^*; *Boc*^−/−^ incisor tooth germs and no significant enamel or dentine deposition, only bony deposits within the pulpal regions of these teeth. However, mandibular incisor and all molar development appeared normal in the double mutant (Fig. 5P–U).

### *Gas1^−/−^*; *Boc^−/−^* mutant mice have lobar HPE

**Fig. 6.**
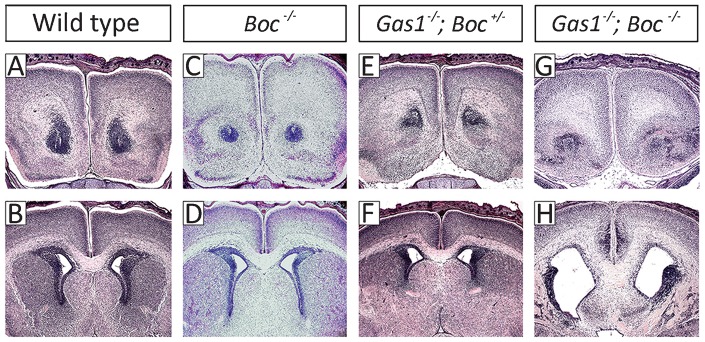
*Gas1^−/−^*; *Boc^−/−^* compound mutants have lobar HPE. Frontal sections through the developing forebrain at E17.5 stained with haematoxylin and eosin. (A,B) Wild-type; (C,D) *Boc^−/−^*; (E,F) *Gas1^−/−^*; *Boc^+/−^* mice all have normal development of the forebrain; (G,H) *Gas1^−/−^*; *Boc^−/−^* mice have separation of the telencephalic vesicles.

The findings that combined loss of *Gas1* and *Boc* function was associated with more severe midline facial defects than those seen in the absence of *Gas1* or *Boc* alone, prompted us to investigate the developing forebrain of these mice (Fig. 6A–H). At E18.5, wild-type and *Boc^−/−^* mice have normal subdivision of the anterior forebrain into left and right telencephalic vesicles, with the corpus callosum extending throughout the dorsal forebrain and an intact diencephalon in the ventral midline (Fig. 6A–D). As expected, gross development of these structures was also normal in *Gas1^−/−^*; *Boc^+/−^* mice, consistent with the presence of microform HPE in these animals (Fig. 6E,F). However, whilst *Gas1^−/−^*; *Boc^−/−^* mice had appropriate telencephalic separation, development of the corpus callosum was disrupted, with a lack of definition, particularly in the midline. In addition, the lateral ventricles were enlarged and patterning of the diencephalon disorganized (Fig. 6G,H). These findings were consistent with the presence of lobar HPE in the absence of both *Gas1* and *Boc* alleles.

### Progressive loss of Shh transduction in the CNS and facial midline in the absence of *Gas1* and *Boc*

**Fig. 7.**
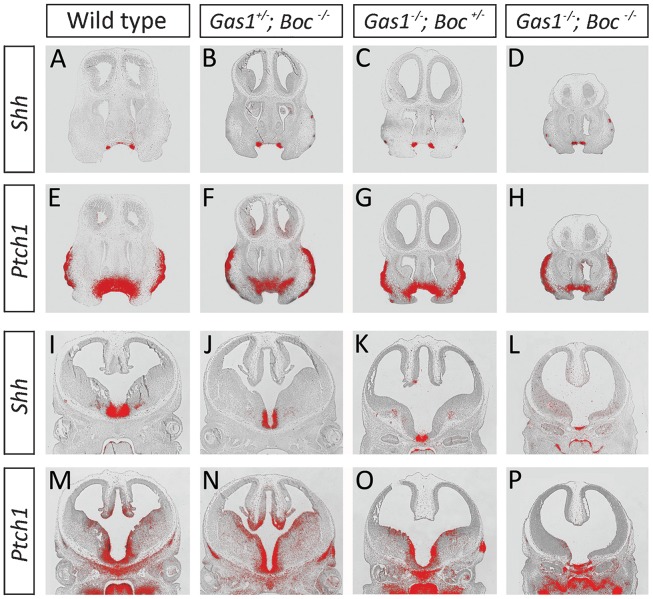
Reduced Shh signaling in the craniofacial midline in the absence of *Gas1* and *Boc* function. Reduced Shh signaling at E12.5 in the facial midline and CNS in the combined absence of *Gas1* and *Boc*. (A–D) *Shh* and (E–H) *Ptch1* expression in the facial midline through the developing maxillary incisor teeth; (I–L) *Shh* and (M–P) *Ptch1* expression in the developing CNS. *Shh* expression is normal in the incisor teeth of all genotypes (A–D), whilst *Ptch1* expression is comparable to wild-type in *Gas1^+/−^*; *Boc^−/−^* mice (E,F). However, *Ptch1* is progressively reduced in the underlying mesenchyme of *Gas1^−/−^*; *Boc^+/−^* and *Gas1^−/−^*; *Boc^−/−^* mice, respectively (G,H). In the ventral region of the developing CNS, *Shh* expression is comparable to wild-type in *Gas1^+/−^*; *Boc^−/−^* mice (I,J), but progressively reduced in *Gas1^−/−^*; *Boc^+/−^* and *Gas1^−/−^*; *Boc^−/−^* mice (K,L). Consistent with the findings of microform HPE in *Gas1^−/−^*; *Boc^+/−^* mice, *Ptch1* expression is normal in the ventral CNS; however, in *Gas1^−/−^*; *Boc^−/−^* mice there is lobar HPE and reduced expression of *Ptch1* ventrally (P).

The identification of lobar HPE associated with a combined loss of *Gas1* and *Boc* function was suggestive of reduced Shh signaling in the facial midline and CNS of these mutants. We therefore further analyzed *Gas1*; *Boc* compound mutant mice by assaying the expression of *Shh* and the downstream transcriptional targets *Ptch1* and *Gli1* (data not shown) at E12.5 (Fig. 7A–P). In the developing facial midline, levels of *Shh* transcription within the early incisor epithelium were comparable across genotypes (Fig. 7A–D). However, whilst *Ptch1* (and *Gli1*, data not shown) demonstrated expression comparable to wild-type in the underlying midline facial mesenchyme of *Gas1^+/−^*; *Boc^−/−^* mutants (Fig. 7E,F), a progressive reduction was seen in *Gas1^−/−^*; *Boc^+/−^* and *Gas1^−/−^*; *Boc^−/−^* mutants, respectively (Fig. 7G,H). These data were suggestive of a direct role for Boc in facilitating Shh transduction during early development of the facial midline. We also analyzed Shh transduction in the midline of the developing CNS. At E12.5, *Gas1^−/−^*; *Boc^−/−^* mice had a reduction in *Shh* transcription in the ventral midline when compared to wild-type, *Gas1^+/−^*; *Boc^−/−^* and *Gas1^−/−^*; *Boc^+/−^* mice (Fig. 7I–L). Consistent with the presence of lobar HPE, this was accompanied by reduced Shh transduction, as demonstrated by the reduced extent of *Ptch1* (and *Gli1*, data not shown) expression in the midline of *Gas1^−/−^*; *Boc^−/−^* mice when compared to the other genotypes (Fig. 7M–P). Therefore, the lobar HPE observed in *Gas1^−/−^*; *Boc^−/−^* mice was secondary to reduced Shh signaling in the early midline of the developing face and CNS, consistent with a role for Gas1 and Boc as Shh co-receptors.

### *Boc* is required for cell survival in the maxillary incisor epithelium in the absence of *Gas1*

**Fig. 8.**
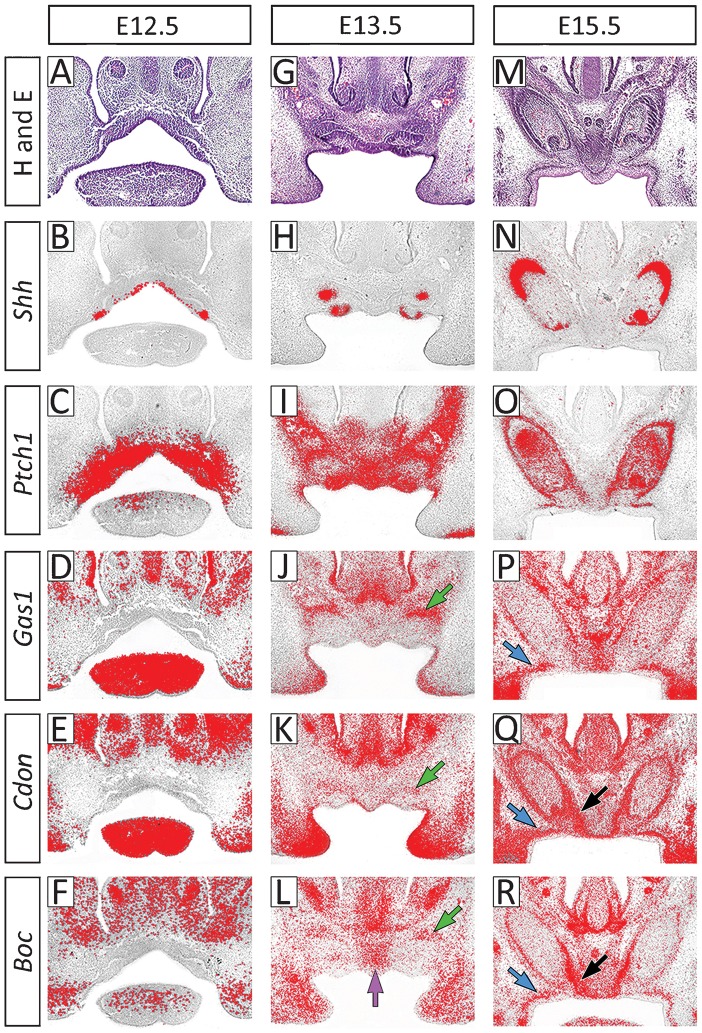
Shh receptor gene expression in the developing maxillary incisor dentition at E12.5, 13.5 and 15.5. (A,G,M) H and E; (B,H,N) *Shh*; (C,I,O) *Ptch1*; (D,J,P) *Gas1*; (E,K,Q) *Cdon*; (F,L,R) *Boc*. Purple arrow indicates *Boc* expression in the midline mesenchyme at E13.5; green arrows indicate *Gas1*, *Cdon* and *Boc* expression in the dental papilla at E13.5; blue arrows indicate *Gas1*, *Cdon* and *Boc* expression in mesenchyme directly below the oral epithelium at E15.5; black arrows indicate *Cdon* and *Boc* expression in the dental lamina at E15.5.

The incisor phenotype observed in the absence of *Gas1* and *Boc* function has not previously been described in association with HPE. In an attempt to further understand why *Gas1* and *Boc* are collectively essential for maxillary incisor development we mapped expression of these genes during this process and compared them with members of the Shh pathway (Fig. 8A–R). At E12.5, during the early stages of incisor development, *Shh* is expressed in the early incisor epithelial thickenings, whilst *Ptch1* expression demarcates pathway activity throughout the midline. At this stage *Gas1*, *Cdon* and *Boc* were all co-expressed in mesenchyme at the peripheral margins of Shh activity (Fig. 8A–F). By E13.5, at the bud stage of incisor development, *Shh* remains localized to the tooth bud epithelium, whilst *Ptch1* is strongly expressed in the epithelium and condensing mesenchymal papilla of these teeth. However, whilst *Gas1*, *Cdon* and *Boc* were all upregulated in the dental papilla of the incisor tooth germs, their expression domains demonstrated some subtle differences. In particular, *Gas1* and *Boc* were expressed in more peripheral regions of the dental papilla destined to form the dental follicle, whilst *Cdon* was localized to that region of the papilla directly adjacent to the epithelium. Interestingly, *Boc* was the only one of these co-receptors to demonstrate upregulation in the midline mesenchyme between the two incisor tooth germs (Fig. 8G–L). At E15.5, during the late cap and early bell stage *Shh* localizes to presecretory ameloblasts within the enamel organ, whilst *Ptch1* expression is seen in the outer enamel epithelium and dental papilla. At this stage, all three co-receptors were expressed in the outer enamel epithelium of the incisor enamel organs, overlapping with *Ptch1*; whilst *Cdon* and *Boc* were also strongly expressed in the dental lamina of these teeth. In addition, all three genes were upregulated in a small domain of mesenchyme situated directly adjacent to the oral epithelium and immediately lateral to the incisors, which in the case of *Gas1* and *Cdon*, became continuous with a region of intense expression in the facial process (Fig. 8M–R). Collectively, these data are consistent with a potential role for Boc in mediating Shh signaling within odontogenic epithelium and mesenchyme during the cap and bell stages of maxillary incisor development.

**Fig. 9.**
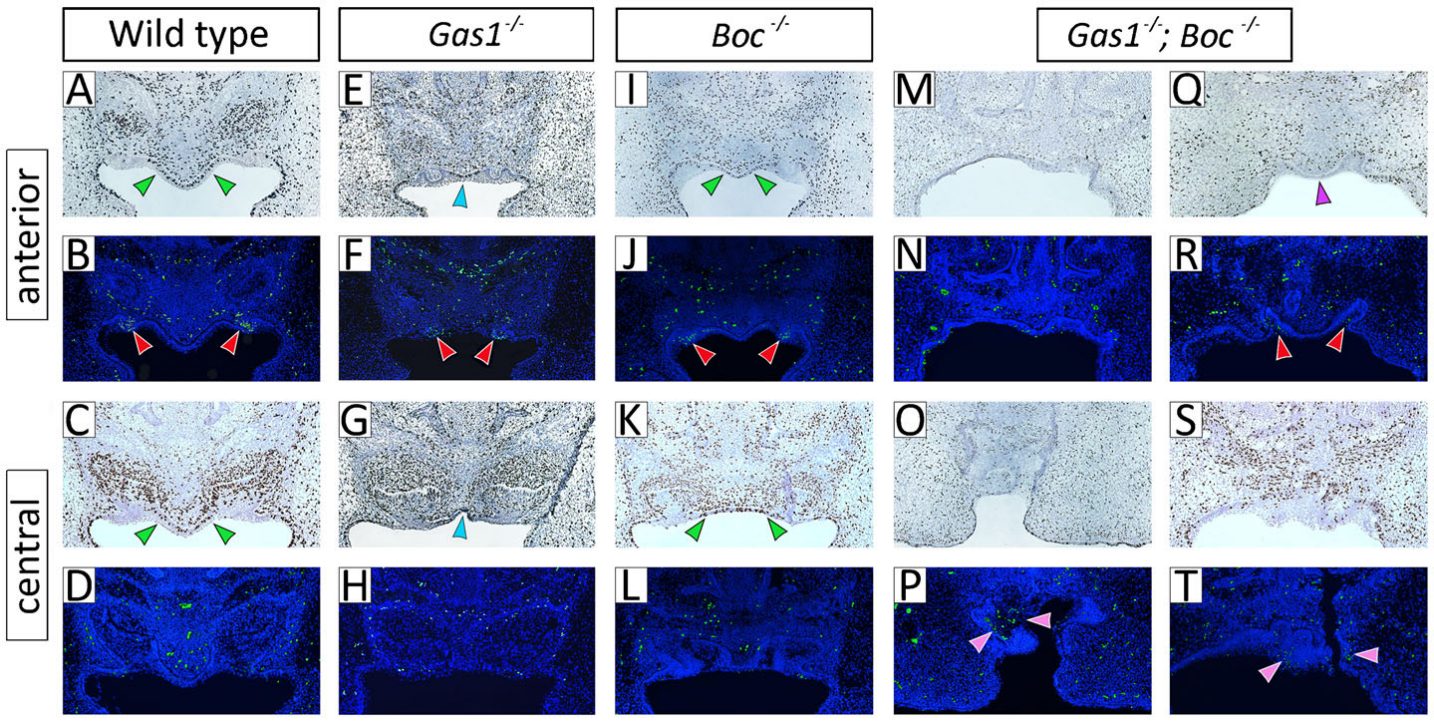
Reduced proliferation in the facial midline and increased cell death in the maxillary incisor epithelium in *Gas1*; *Boc* compound mutant mice. Frontal sections through the developing maxillary incisor region at E14.5 in (A–D) wild-type; (E–H) *Gas1^−/−^*; (I–L) *Boc^−/−^*; and (M–T) *Gas1^−/−^*; *Boc^−/−^* mutant mice. The anterior and central regions of the incisor tooth buds have been BrdU labeled for cell proliferation (upper panels) and TUNEL stained for apoptosis (lower panels), respectively. A clear region of epithelial proliferation exists between the developing incisors in wild-type and *Boc^−/−^* mice (green arrowheads in panels A,C and I,K, respectively), which is absent in the *Gas1^−/−^*; *Boc^−/−^* (purple arrowhead in panel Q). In *Gas1^−/−^* mice, there is evidence of some proliferation in the midline epithelium but this region is constricted in comparison to wild-type and Boc^−/−^ mice (light blue arrowheads in panels E,G; note in this example there are two individual incisor tooth germs). In addition, the *Gas1^−/−^*; *Boc^−/−^* mutant has regions of epithelial apoptosis within the body of its disrupted tooth germ (pink arrowheads panel in P,T), which were never seen in either wild-type or *Boc^−/−^* incisors at E14.5. Note the discreet areas of epithelial apoptosis present in the anterior region of wild-type, *Gas1^−/−^*, *Boc^−/−^* and *Gas1^−/−^*; *Boc^−/−^* incisors (red arrowheads in panel B,F,J,R, respectively).

The disorganized architecture and ultimate arrest of maxillary incisor tooth development in *Gas1^−/−^*; *Boc^−/−^* mice was suggestive of defective cell cycle regulation within these tooth germs. We therefore analyzed cell proliferation and survival in wild-type, *Gas1^−/−^*, *Boc^−/−^* and *Gas1^−/−^*; *Boc^−/−^* incisors at E14.5 using BrdU and TUNEL staining, respectively (Fig. 9A–T). In wild-type mice, a clear region of epithelial cell proliferation was present in the anterior midline of the premaxilla, which was also identifiable more posteriorly in the region between the incisor tooth germs (Fig. 9A,C). Localized regions of apoptosis were seen in the anterior-most epithelium of the wild-type maxillary incisors, although further posteriorly no significant cell death was visible within the body of the developing cap itself (Fig. 9B,D). These patterns of cell proliferation and apoptosis were also seen in the developing maxillary incisor region of *Gas1^−/−^* and *Boc^−/−^* mice, although in the *Gas1* mutant there was evidence of constriction and a lack of development across the midline (Fig. 9E–L). However, in *Gas1^−/−^*; *Boc^−/−^* mice there was no obvious proliferation within the midline oral epithelium of the premaxilla, although small regions of apoptosis were visible in the anterior region of the mutant tooth germs. Significantly, in more posterior regions, there was no clear demarcation of any oral epithelium between two separate tooth germs, just a mass of poorly organized odontogenic epithelium. Within this epithelium and the underlying mesenchyme there was evidence of proliferation, particularly in those regions of epithelium adjacent to the mesenchyme. However, more centrally there was little proliferation, just extensive regions of apoptosis (Fig. 9L–P). Therefore, an absence of *Gas1* and *Boc* function was associated with a lack of proliferation in the midline oral epithelium of the premaxilla and increased cell death within the disorganized epithelium of the incisor itself.

## DISCUSSION

We report here the craniofacial features of mice lacking function of the Shh co-receptors Gas1, (Cdon) and Boc, focusing on the effects of combined Gas1 and Boc loss-of-function. Importantly, we find that loss of *Boc* in a *Gas1* mutant background significantly increases severity of the craniofacial defects that are seen in the absence of *Gas1* alone. *Gas1^−/−^*; *Boc^−/−^* mice have lobar HPE, associated with disruption of the corpus callosum, disorganization within the diencephalon, unilateral cleft lip and palate, midline clefting within the posterior third of the tongue and severely disrupted maxillary incisor development. Given the known etiological heterogeneity of HPE, these findings suggest that *BOC* represents a potential modifier locus for HPE in human populations. Interestingly, the human *BOC* gene is known to be situated on chromosome 3q13.2 and deletions of 3q13.1q13.3 or 3q13.2q21.3 have both previously been associated with agenesis of the corpus callosum and abnormal facial features (Genuardi et al., 1994; Lawson-Yuen et al., 2006). Moreover, HPE has also been described in one case of 3q13q21 deletion (Arai et al., 1982). Whilst deletions of the proximal long arm of chromosome 3 are rare, abnormalities associated with the CNS and facial dysmorphology are consistent features amongst those cases that have been described (Molin et al., 2012; Shuvarikov et al., 2013).

Previous work has shown evidence of specific requirements for *Gas1*, *Cdon* and *Boc* but also redundancy during the regulation of Shh signaling in different developmental contexts. In the chick neural tube, over-expression experiments have demonstrated equivalence between these co-receptors in the promotion of Shh-dependent specification of ventral neural precursors (Allen et al., 2011; Allen et al., 2007; Martinelli and Fan, 2007; Tenzen et al., 2006); although *Gas1* is also capable of attenuating the response to Shh in cultured mandibular mesenchyme and dorsal somite (Cobourne et al., 2004; Lee et al., 2001a). A loss of *Gas1* (Allen et al., 2011; Allen et al., 2007; Martinelli and Fan, 2007) or *Cdon* (Allen et al., 2011; Tenzen et al., 2006) produces ventral patterning defects in the mouse neural tube, but this region is essentially normal in *Boc^−/−^* mice (Allen et al., 2011). In contrast, mice lacking the collective function of *Gas1*, *Cdon* and *Boc* effectively lack all Shh signaling except for some very early transient activity and as a result, have complete absence of Shh-dependent neural progenitors, heart-looping defects and alobar HPE (Allen et al., 2011). The combinatorial loss of *Gas1*, *Cdon* or *Boc* leads to a progressive worsening of Shh-dependent neural patterning in compound mutants (Allen et al., 2011; Allen et al., 2007). The most severe disruption is seen with a loss of either *Cdon* or *Boc* in the absence of *Gas1*; however, *Cdon^−/−^*; *Boc^−/−^* embryos also have more severe defects than are seen in any single mutants (Allen et al., 2011; Allen et al., 2007). In the developing limb bud there are also different requirements for these co-receptors. *Gas1* mutant mice have disrupted digit specification with syndactyly or variable absence of digit 2 or 3 in both forelimb and hindlimb (Allen et al., 2007; Liu et al., 2002; Martinelli and Fan, 2007); but the limbs of *Cdon* and *Boc* single and compound mutants are essentially normal (Allen et al., 2011; Zhang et al., 2011). Interestingly, the loss of both *Cdon* or a single *Boc* allele in a *Gas1^−/−^* background does not exacerbate the limb phenotype; however, a loss of both *Boc* alleles in the absence of *Gas1* results in significant worsening (a lack of digit 2 is combined with fusion of digits 3 and 4) (Allen et al., 2011).

The craniofacial defects associated with loss of *Gas1*, *Cdon* and *Boc* are also variable and background-dependent (supplementary material Table S1). *Gas1* or *Cdon* absence primarily causes microform HPE, although *Cdon* mutant mice on a congenic C57/BL/6 background have semilobar HPE (Zhang et al., 2006). These HPE microforms display subtle differences in both their features and penetrance. On a 129sv/C57BL/6 background, *Cdon^−/−^* mice have numerous anomalies associated with the premaxilla, whilst in *Gas1^−/−^* mice this region is less affected, but there is a higher incidence of cleft palate (Cole and Krauss, 2003; Seppala et al., 2007). The severity of HPE does increase with the loss of combined alleles; *Gas1^−/−^*; *Cdon^−/−^* mutants have a severe form of alobar HPE, which includes fusion of the nasal processes and absence of maxillary and mandibular skeletal elements (Fig. 3M–O) (Allen et al., 2007), whilst *Cdon^−/−^*; *Boc^−/−^* mice on a Cdon-resistant background have lobar HPE with more severe craniofacial abnormalities (Zhang et al., 2011). Here, we now show that *Gas1* and *Boc* are also required collectively for normal development of the craniofacial midline, including the early forebrain and that the phenotype becomes progressively worse in the absence of both alleles.

Gas1, Cdon and Boc all bind Shh with high affinity and can each form distinct receptor complexes with Ptch1 (Izzi et al., 2011; Lee et al., 2001a; Martinelli and Fan, 2007; McLellan et al., 2008; Okada et al., 2006; Tenzen et al., 2006). These interactions are essential for Shh transduction in mammalian systems, indicating that the binding of Shh to Ptch1 alone is not sufficient to activate transduction (Allen et al., 2011). A number of specific functions have been identified for these co-receptors at the cellular level within the developing CNS, including Shh-mediated commissural axonal guidance, neural progenitor specification, motor neuron maintenance and Shh-dependent proliferation of cerebellar granule neuron precursor cells (Allen et al., 2011; Izzi et al., 2011; Okada et al., 2006). Here, we identify a role for *Boc* in promoting cell survival in odontogenic epithelium of the developing maxillary incisor in the absence of *Gas1* function. Interestingly, patterns of apoptosis within the developing incisors were grossly normal in *Gas1* and *Boc* single mutant mice, the increased levels seen in compound mutant mice demonstrating that Gas1 and Boc1 each play significantly redundant roles in preventing apoptosis (and promoting proliferation) in this region. Shh signaling is known to be important for normal tooth development, with early signaling from epithelium to mesenchyme required for initiation (Cobourne et al., 2001; Sarkar et al., 2000), inter-epithelial signals contributing to normal morphogenesis of the tooth germ (Dassule et al., 2000; Gritli-Linde et al., 2002) and a lack of signal transduction in cranial neural crest cells leading to an absence or arrest of tooth development (Jeong et al., 2004). Appropriate regulation of signaling levels is also important; with increased epithelial transcription capable of arresting all tooth development secondary to a lack of proliferation (Cobourne et al., 2009). These and other studies have firmly established a link between Shh signaling and cell proliferation within the tooth germ; however, a role in cell survival has only previously been demonstrated in vitro, albeit in both squamate and mammalian teeth (Cobourne et al., 2001; Handrigan and Richman, 2010). More recently, both PTCH1 and CDON have been identified as SHH-dependence receptors, able to induce apoptosis in the absence of SHH ligand; however, there is currently no evidence that BOC (or indeed, GAS1) have similar characteristics (Delloye-Bourgeois et al., 2013; Mille et al., 2009; Thibert et al., 2003). It is also interesting to note the link between Shh signaling and later histodifferentiation in the developing tooth. In the combined absence of *Gas1* and *Boc* there was little evidence of ameloblast or odontoblast differentiation, which may be secondary to the cell death seen at the disrupted cap stage; however, Shh also mediates matrix secretion in the squamate tooth germ and conditional loss of Shh in odontogenic epithelium results in abnormal polarization of both ameloblast and odontoblast cells (Dassule et al., 2000).

It is difficult to reconcile the subtle differences in expression domains that exist between *Gas1*, *Cdon* and *Boc* in the developing maxillary incisor region and their different requirements during normal development of these teeth. This is similar to the neural tube and limb bud, where differences in the transcriptional and protein domains of these co-receptors do not correlate directly with phenotype (Allen et al., 2011; Martinelli and Fan, 2007; Tenzen et al., 2006). In the incisors, expression of these co-receptors is first seen at the bud stage in the dental papilla, but then localizes to the outer enamel epithelium and dental lamina by the late cap stage. This suggests that they are involved in mediating both short and long-range Shh signaling during incisor development, but as the phenotype of different combinations of compound mutants demonstrates, with varying influence. *Gas1^−/−^* and *Cdon^−/−^* mice have SMMCI with incomplete penetrance, whilst *Boc^−/−^* incisors are normal. *Gas1*; *Cdon* compound mutants lack a premaxilla and are therefore not informative with regard to maxillary incisor formation; however, *Gas1^+/−^*; *Cdon^+/−^* and *Gas1^−/−^*; *Cdon^+/−^* mice have SMMCI with increasing severity, although gross tissue organization within these teeth is normal. *Cdon^−/−^*; *Boc^−/−^* mice have an absence of the maxillary incisors and as we report here, combined loss of *Gas1* and *Boc* function results in only rudimentary maxillary incisor development (supplementary material Fig. S1). Therefore it would appear that there is a hierarchy of influence amongst these co-receptors during development within the premaxillary region, with loss of *Gas1* and *Boc* resulting in an absence of appropriate histodifferentiation of the cap stage tooth germ.

The analysis of Hedgehog co-receptor function has demonstrated an important role for these proteins in the etiology of HPE and provided insight into the phenotypic heterogeneity that characterizes this malformation sequence. Here, we demonstrate a unique form of alobar HPE in mice lacking Gas1 and Boc function. These mice have cleft lip and palate, clefting within the pharyngeal tongue and maxillary incisor development that arrests in association with epithelial apoptosis. Collectively, our data provide evidence for *BOC* as a potential modifier for HPE in human populations.

## Supplementary Material

Supplementary Material
