# Supplementary material for: Boc modifies the spectrum of holoprosencephaly in the absence of Gas1 function
Source: Biol Open. 2014 Jul 25;3(8):728–40. doi: 10.1242/bio.20147989 (PMC4133726; doi:10.1242/bio.20147989)
Supplement: Supplementary Material [file supp_3_8_728__index.html]

Boc modifies the spectrum of holoprosencephaly in the absence of Gas1 function — Boc modifies the spectrum of holoprosencephaly in the absence of Gas1 function — Supplementary Material 

# *Boc* modifies the spectrum of holoprosencephaly in the absence of *Gas1* function

## bio.20147989 Supplementary Material

**Files in this Data Supplement:**

- Supplementary Material - Maisa Seppala et al. doi: 10.1242/bio.20147989
