# Supplementary material for: Boc modifies the spectrum of holoprosencephaly in the absence of Gas1 function
Source: Biol Open. 2014 Jul 25;3(8):728–40. doi: 10.1242/bio.20147989 (PMC4133726; doi:10.1242/bio.20147989)
Supplement: Supplementary Material [file supp_bio.20147989_bio.20147989-s1.pdf]

Biology Open (2014) 000, 1–13 doi:10.1242/bio.20147989

Biology Open

Supplementary Material

Maisa Seppala et al. doi: 10.1242/bio.20147989

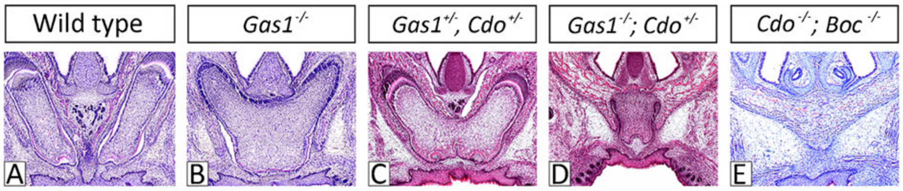

Fig. S1. Gradation of incisor phenotype in *Gas1*; *Cdo* and *Boc* compound mutants. Frontal sections through the developing maxillary incisors at E17.5 stained with haematoxylin and eosin. (A) In wild-type mice, the paired maxillary incisors are at the crown stage of development with normal hard tissue formation. (B) In *Gas1*$^{-/-}$; (C) *Gas1*$^{+/-}$; *Cdo*$^{+/-}$; (D) *Gas1*$^{-/-}$; *Cdo*$^{+/-}$ mice there is SMMCI with increasing severity, although normal hard tissue formation is present in these teeth. (E) *Cdo*$^{-/-}$; *Boc*$^{-/-}$ mice have an absence of incisor formation.

Table S1. Strain-dependent severity of HPE in *Gas1*, *Cdo* and *Boc* mutant mice

Mutant allele	Background	Craniofacial phenotype	Reference
Gas1	129sv/CD1	Normal	Lee et al., 2001b; Liu et al., 2001
	129sv/C57BL/6	Microform HPE, including maxillary incisor fusion, premaxillary synostosis, cleft palate	Seppala et al., 2007
Cdo	129S6	Microform HPE (30% penetrance)	Hong and Krauss, 2012
	129sv/C57BL/6	Microform HPE (95% penetrance), including philtral dysgenesis, maxillary incisor agenesis or fusion, premaxillary fusion or agenesis	Cole and Krauss, 2003
	129sv	Microform HPE (50%) penetrance	Zhang et al., 2006
	Congenic C57BL/6	Semilobar HPE, cebocephalic face (single nostril, ocular hypotelorism, maxillary hypoplasia) (80% penetrance)	Zhang et al., 2006
Boc	Mixed 129B6/Congenic 129	Normal	Okada et al., 2006; Zhang et al., 2011
